# Supplementary material for: Species-specific, accession-specific, and common responses of foliar phytohormones and morphological traits to drought and herbivory
Source: BMC Plant Biol. 2026 May 27;26:923. doi: 10.1186/s12870-026-09091-8 (PMC13217867; doi:10.1186/s12870-026-09091-8)
Supplement: Supplementary file 1 — Supplementary Material 1. [file 12870_2026_9091_MOESM1_ESM.pdf]

Species-specific, accession-specific, and common responses of foliar phytohormones and morphological traits to drought and herbivory

Xue Xiao, Kruthika Sen Aragam, Andrea Bräutigam, Thomas Dussarrat, Selina Gaar, Maximilian Hanusch, Robin Heinen, Marvin Hildebrandt, Ruth Jakobs, Robert R. Junker, Radhika Keshan, Judit V. Mendoza Servín, Elikplim A. Setordjie, Yonca B. Seymen, Anke Steppuhn, Sybille B. Unsicker, Nicole M. van Dam, Baris Weber, Sarah K. Weirauch, Wolfgang W. Weisser, Dominik Ziaja, Jörg-Peter Schnitzler, J. Barbro Winkler, Caroline Müller

Table S1. Time schedule of experiments

	Tanacetum vulgare	*Solanum dulcamara*	*Populus nigra*
Plant cuttings preparation	18/09/2024	27/01/2025	12/10/2024
Transport plants to Munich	18/09/2024	17/02/2025	16/12/2024
Transfer plants into climate chambers	04/12/2024	03/03/2025	16/06/2025
[1]EK1+2: onset of drought treatment (defined as the day after the last uniform watering (day 0))	13/01/2025	11/03/2025	23/06/2025
[2]EK3+4: onset of drought treatment	14/01/2025	12/03/2025	24/06/2025
Set larvae on leaf mix for adaptation	21/01/2025	18/03/2025	30/06/2025
EK1+2: Herbivory treatment	23/01/2025	20/03/2025	04/07/2025
EK3+4: Herbivory treatment	24/01/2025	21/03/2025	05/07/2025
EK 1+2: VOC collection	26/01/2025	23/03/2025	07/07/2025
EK 3+4: VOC collection	27/01/2025	24/03/2025	08/07/2025
EK1+2: Herbivore removal, shoot harvest, phenotyping	27/01/2025	24/03/2025	08/07/2025
EK3+4: Herbivore removal, shoot harvest, phenotyping	28/01/2025	25/03/2025	09/07/2025
EK1+2: Root harvest	29/01/2025	26/03/2025	08/07/2025
EK3+4: Root harvest	30/01/2025	27/03/2025	09/07/2025

[1]EK1+2: main chambers one and two; [2]EK3+4: main chambers three and four

Table S2. Origin of accessions of *Tanacetum vulgare* individuals and their chemotypes

Accession	Chemotype[1]	Coordinates (Country)	Collection date in field (collector)
M6	α-chrysanthenyl acetate (6_22)	51°58.913"N 8°27.629"E (Germany)	29/01/2019 (Elisabeth J. Eilers, Ruth Jakobs)
	β-carvylacetate (6_27)		
M9	α-thujone (9_19)	51°58.662"N 8°27.273" E(Germany)	29/01/2019 (Elisabeth J. Eilers, Ruth Jakobs)
	α-thujone, β-thujone (9_35)		
	artemisia ketone (9_77)		
M11	β-thujone (11_7)	51°58.648"N 8°27.243"E (Germany)	29/01/2019 (Elisabeth J. Eilers, Ruth Jakobs)
M16	artemisia ketone, artemisyl acetate (16_40)	51°58.601"N 8°27.173"E (Germany)	29/01/2019 (Elisabeth J. Eilers, Ruth Jakobs)
	α-thujone, β-thujone (16_175)		
M18	camphene, cis-verbenol acetate (18_11)	51°59.031"N 8°28.302"E (Germany)	29/01/2019 (Elisabeth J. Eilers, Ruth Jakobs)
	(*Z*)-myroxide, santolina triene, artemisyl acetate (18_81)		
M22	artemisia ketone (22_5)	51°58.994"N 8°28.388"E (Germany)	29/01/2019 (Elisabeth J. Eilers, Ruth Jakobs)
	(*Z*)-myroxide, santolina triene, artemisyl acetate (22_16)		

Per accession (maternal origin) and offspring chemotype combination, 16 clones were produced from the offspring.

[1]The dominant leaf terpenoid(s) is (are) given. Chemotypes were determined in the seedling stage of the offspring plants

Table S3. Origin of accessions of *Solanum dulcamara* individuals

**Accession**	**Coordinates (Country)**	**Collection date in field (collector)**	**Selfed offspring generation (yes/no)**
SIE/A27	52°16'53.6"N 13°11'17.4"E (Germany)	13/10/2013 (Tobias Lortzing)	yes
EBB/16	48°50'52.1"N 9°35'48.8"E (Germany)	22/10/2020 (Anke Stepp-uhn)	no
EBB/18	48°51'11.3"N 9°35'36.9"E (Germany)	22/06/2021 (Kruthika S Aragam)	no
OW09	51°51'36.3"N 5°54'03.5"E (Netherlands)	Autumn 2012 and 2013 (Qian Zhang, et al.)	no
VW08	51°50'58.6"N 4°04'34.3"E (Netherlands)	Autumn 2012 and 2013 (Qian Zhang, et al.)	no
TW12	53°07'17.5"N 4°47'13.6"E (Netherlands)	Autumn 2012 and 2013 (Qian Zhang, et al.)	no

Per accession, 32 clones were produced.

Table S4. Origin of accessions of *Populus nigra* individuals

**Accession**	**Sex**	**Coordinates (Germany)** Topographic map sheet number - Municipality code – object # in this area; City of Marbach am Neckar[1]	**Collection date in field (collector)**
R14	Female	7221-116- 01	2012-2013 by „Arbeitskreis zur Erhaltung der Neckarschwarzpappel, Stiftung Energie & Klimaschutz Badenn-Württemberg“[1],
R19	Female	7421-116- 06	
R20	Female	7421-116- 07	
R25	Male	7019-118- 01	
R30	Male	7021-118- 04	
R32	Male	7021-118- 11	

Per accession, 16 clones were produced.

[1]https://www.energie-klimaschutz.de/wp-content/uploads/2015/01/Neckarschwarzpappel-Projektinformation-II.pdf

Table S5A. Watering regime (mL water/pot) for *Tanacetum vulgare* in the different treatment groups. The onset of drought was defined as the first day after the last uniform watering (13/01/2025 = day 0 for EK1 and EK2, 14.01.2025 = day 0 for EK3 and EK4). When two dates are listed, the first refers to watering in EK1 and EK2 and the second to watering in EK3 and EK4.

		**Treatment groups**			
**Date**	**Days of drought**	**Control**	**Herbivory**	**Drought**	**Combined**
06/01/2025	day-7 / day-8	200	200	200	200
09/01/2025*	day -4 / day-5	200	200	200	200
12/01/2025	day -1 / day-2	200	200	200	200
15.,16/01/2025	day +2	200	200	100[1]	100[1]
17.,18/01/2025	day +4	200	200	100	100
19.,20/01/2025	day +6	200	200	100	100
21.,22.01/2025	day +8	200	200	100	100
23.,24.01/2025	day +10	200	200	100	100
24.,26/01/2025	day +12	200	200	100	100
	**Total**	**1800**	**1800**	**1200**	**1200**

*including 0.5% Hakaphos rot (Compo, GmbH, Germany) fertilizer

[1] 10 pots in drought and 13 pots in the combined treatment, respectively, received additional 50 mL to avoid wilting.

Table S5B. Watering regime (mL water/pot) for *Solanum dulcamara* in the different treatment groups. The onset of drought was defined as the first day after the last uniform watering (11/03/2025 = day 0 for EK1 and EK2, 12.03.2025 = day 0 for EK3 and EK4). When two dates are listed, the first refers to watering in EK1 and EK2 and the second to watering in EK3 and EK4.

		**Treatment groups**			
**Date**	**Days of drought**	**Control**	**Herbivory**	**Drought**	**Combined**
06.,07/03/2025	day -5	100	100	100	100
08.,09/03/2025	day -3*	100[1]	100	100	100[1]
10.,11/03/2025	day -1	100	100	100	100
12.,13/03/2025	day +1	100	100		
14.,15/03/2025	day +3	200	200	100	100
16.,17/03/2025	day +5	200	200	100	100
18.,19/03/2025	day +7	200	200	100[2]	100[2]
20.,21/03/2025	day +9	300	300	150[2]	150[2]
22.,23/03/2025	day + 11	300	300	150[2]	150[2]
	**Total**	**1600**	**1600**	**900**	**900**

[*]including 0.5% Hakaphos rot (Compo, GmbH, Germany) fertilizer

[1] Due to a malfunction in the automatic irrigation system, 12 plants in each of the control and combined treatment groups received 200 mL of water.

[2] 5 plants in the drought and 3 plants in the combined treatment received 50 mL less water due to their size.

Table S5C. Watering regime (mL water/pot) for *Populus nigra* in the different treatment groups. The onset of drought was defined as the first day after the last uniform watering (26.,27/06/2025 = day 0 for EK1 and EK2, 27/06/2025 = day 0 for EK3 and EK4). When two dates are listed, the first refers to watering in EK1 and EK2 and the second to watering in EK3 and EK4.

		**Treatment groups**			
**Date**	**Days of drought**	**Control**	**Herbivory**	**Drought**	**Combined**
18/06/2025	day -9	200	200	200	200
20/06/2025	day -7	200	200	200	200
23/06/2025	day -4	200	200	200	200
26.,27/06/2025	day 0	200	200	100	100
29.,30/06/2025	day +3	200	200	100	100
01.,02/07/2025	day +5	200	200	100	100
03.,04/07/2025	day +7	300	300	150	150
	**Total**	**1500**	**1500**	**1050**	**1050**

Table S6A: Soil water content (SWC, vol-%) in *Tanacetum vulgare* pots, determined using a soil moisture meter (HH2) connected to a ThetaProbe (ML2x; Delta-T Devices. EKs denote the experimental chambers in which measurements were performed. Values are presented as mean ± standard deviation (SD) and standard error (SE). When four chambers were measured, *n* = 48 per treatment; when two chambers were measured, *n* = 24 per treatment.

Date	Day of drought	SWC	Treatment				EKs
			Control (vol-%)	Herbivory (vol-%)	Drought (vol-%)	Combined (vol-%)	
07/01/2025	day -6	mean	28.3	26.9	26.9	25.9	EK 1-4
		SD	11.2	10.3	11.0	9.7	
		SE	1.7	1.6	1.7	1.5	
13/01/2025	day 0	mean	25.9	26.6	25.1	25.8	EK 1-4
		SD	14.0	11.7	11.1	10.6	
		SE	2.0	1.7	1.6	1.5	
16/01/2025	day + 3	mean			11.9	12.5	EK 1-4
		SD			5.7	6.8	
		SE			0.8	1.0	
17/01/2025	day +4	mean			9.2	9.6	EK 3+4
		SD			4.7	5.8	
		SE			1.0	1.2	
20/01/2025	day +7	mean	30.4	27.9	11.6	12.6	EK 1+2
		SD	14.2	10.2	4.0	5.8	
		SE	2.9	2.1	0.8	1.2	
21/01/2025	day +8	mean	26.3	27.7	13.2	12.5	EK 3+4
		SD	11.8	12.8	8.5	8.3	
		SE	2.4	2.6	1.7	1.7	
22/01/2025	day +9	mean			11.1	11.3	EK 1+2
		SD			5.1	5.5	
		SE			1.0	1.1	
23/01/2025	day +10	mean			11.7	11.2	EK 3+4
		SD			6.3	6.6	
		SE			1.3	1.3	

Table S6B: Soil water content (SWC, vol-%) in *Solanum dulcamara* pots, determined using a soil moisture meter (HH2) connected to a ThetaProbe (ML2x; Delta-T Devices). Values are presented as mean ± standard deviation (SD) and standard error (SE), n = 48 per treatment. EKs denote the experimental chambers in which measurements were performed. When two dates are listed, the first refers to watering in EK1 and EK2 and the second to watering in EK3 and EK4.

Date	Day of drought	SWC	Treatment				EKs
			Control	Herbivory	Drought	Combined	
			vol-%	vol-%	vol-%	vol-%	
06.,07/03/2025	day -5	mean	39.2	39.1	38.0	39.1	EK 1-4
		SD	5.8	5.5	4.9	6.6	
		SE	0.8	0.8	0.7	1.0	
11.,12/03/2025	day 0	mean	30.2	31.8	30.0	32.0	EK 1-4
		SD	8.1	8.8	8.7	8.6	
		SE	1.2	1.3	1.3	1.2	
13.,14/03/2025	day +2	mean	33.6	34.5	22.4	24.3	EK 1-4
		SD	7.6	8.9	9.4	8.9	
		SE	1.1	1.3	1.4	1.3	
17.,18/03/2025	day +6	mean	35.8	35.4	19.2	19.5	EK 1-4
		SD	8.1	8.9	9.9	9.5	
		SE	1.2	1.3	1.4	1.4	
19.,20/03/2025	day +8	mean	35.9	35.6	18.6	18.0	EK 1-4
		SD	7.9	8.5	10.2	8.8	
		SE	1.1	1.2	1.5	1.3	

Table S6C: Soil water content (SWC, vol-%) in *Populus nigra* pots, determined using a soil moisture meter (HH2) connected to a ThetaProbe (ML2x; Delta-T Devices). Values are presented as mean ± standard deviation (SD) and standard error (SE), *n* = 48 per treatment. EKs denote the experimental chambers in which measurements were performed. When two dates are listed, the first refers to watering in EK1 and EK2 and the second to watering in EK3 and EK4.

Date	Day of drought	SWC	Treatment Control	Herbivory	Drought	Combined	EKS
			vol-%	vol-%	vol-%	vol-%	
22.,23/06/2025	day -5	mean	23.9	18.9	19.6	20.6	EK 1-4
		SD	12.9	7.6	7.8	8.9	
		SE	1.9	1.1	1.1	1.3	
26.,27/06/2025	day 0	mean	30.6	24.7	18.9	21.1	EK 1-4
		SD	11.0	8.6	6.7	9.7	
		SE	1.6	1.2	1.0	1.4	
02.,03/07/2025	day +6	mean	27.1	23.8	12.3	13.9	EK 1-4
		SD	12.2	8.8	4.3	7.1	
		SE	1.8	1.3	0.6	1.0	
07.,08/07/2025	day +10	mean	26.6	21.5	9.1	10.2	EK 1-4
		SD	11.7	9.5	2.5	6.0	
		SE	1.7	1.4	0.4	0.9	

Table S7. Means (standard deviations) of phenotypic plasticity (measured as RDPI[1]) of traits of *Populus nigra* of different sex, exposed to different challenges (combined: drought and herbivory).

Trait	Treatment	Female df = 12	Male df = 12	
JA concentration	Herbivory	0.656 (0.071)	0.705 (0.053)	
	Drought	0.323 (0.052)	0.300 (0.063)	
	Combined	0.479 (0.083)	0.510 (0.073)	
JA-Ile concentration	Herbivory	0.487 (0.102)	0.558 (0.075)	
	Drought	0.372 (0.086)	0.513 (0.079)	
	Combined	0.337 (0.068)	0.493 (0.061)	
SA concentration	Herbivory	0.266 (0.044)	0.161 (0.035)	Sex:
	Drought	0.367 (0.061)	0.193 (0.037)	$\chi^2 = 5.57$,
	Combined	0.272 (0.049)	0.254 (0.061)	$p = 0.02$
ABA concentration	Herbivory	0.406 (0.057)	0.411 (0.077)	
	Drought	0.856 (0.055)	0.797 (0.057)	
	Combined	0.819 (0.064)	0.822 (0.042)	
IAA concentration	Herbivory	0.189 (0.045)	0.190 (0.033)	
	Drought	0.276 (0.049)	0.149 (0.031)	
	Combined	0.263 (0.059)	0.166 (0.032)	
Height	Herbivory	0.047 (0.013)	0.056 (0.011)	
	Drought	0.056 (0.014)	0.093 (0.016)	
	Combined	0.067 (0.011)	0.065 (0.015)	
Leaf number	Herbivory	0.057 (0.019)	0.067 (0.012)	
	Drought	0.085 (0.025)	0.064 (0.016)	
	Combined	0.094 (0.030)	0.102 (0.018)	
Total leaf dry mass	Herbivory	0.091 (0.023)	0.069 (0.023)	
	Drought	0.128 (0.026)	0.103 (0.026)	
	Combined	0.122 (0.018)	0.089 (0.020)	
Total stem dry mass	Herbivory	0.136 (0.028)	0.091 (0.023)	
	Drought	0.092 (0.016)	0.136 (0.036)	
	Combined	0.113 (0.012)	0.111 (0.026)	
Total aboveground dry mass	Herbivory	0.104 (0.021)	0.068 (0.021)	
	Drought	0.096 (0.019)	0.097 (0.031)	
	Combined	0.095 (0.016)	0.061 (0.020)	
Root dry mass	Herbivory	0.104 (0.020)	0.098 (0.024)	
	Drought	0.119 (0.023)	0.150 (0.035)	
	Combined	0.093 (0.022)	0.160 (0.025)	
Total dry mass	Herbivory	0.092 (0.018)	0.077 (0.021)	
	Drought	0.085 (0.017)	0.113 (0.033)	
	Combined	0.077 (0.016)	0.087 (0.021)	
Root shoot ratio	Herbivory	0.090 (0.023)	0.059 (0.015)	
	Drought	0.104 (0.032)	0.073 (0.017)	
	Combined	0.103 (0.024)	0.128 (0.026)	
Leaf water content	Herbivory	0.016 (0.003)	0.031 (0.010)	Sex:
	Drought	0.026 (0.006)	0.029 (0.005)	$\chi^2 = 3.59$,
	Combined	0.036 (0.008)	0.059 (0.018)	$p = 0.058$
Stem specific density	Herbivory	0.165 (0.028)	0.216 (0.047)	
	Drought	0.178 (0.033)	0.209 (0.041)	
	Combined	0.157 (0.043)	0.108 (0.028)	

[1]RDPI: $(|x_c - x_s|)/(|x_c + x_s|)$ where X_c and X_s represent the different trait values of clones kept under control (c) and challenge (s) conditions.

Table S8: Global goodness-of-fit of each structure equation model (SEM)

Species	SEM	Fisher' C	*p* value	*df*
Tanacetume vulgare	Treatment and accession	59.61	*0.09*	46
Solanum dulcamara	Treatment and accession	70.76	*0.09*	56
Populus nigra	Treatment and genotype	80.05	0.66	86
	Treatment and sex	91.14	**0.01**	66

Significant *p*-values of ($p \leq 0.05$) are highlighted in bold, marginally significant ($p < 0.1$) in italics.

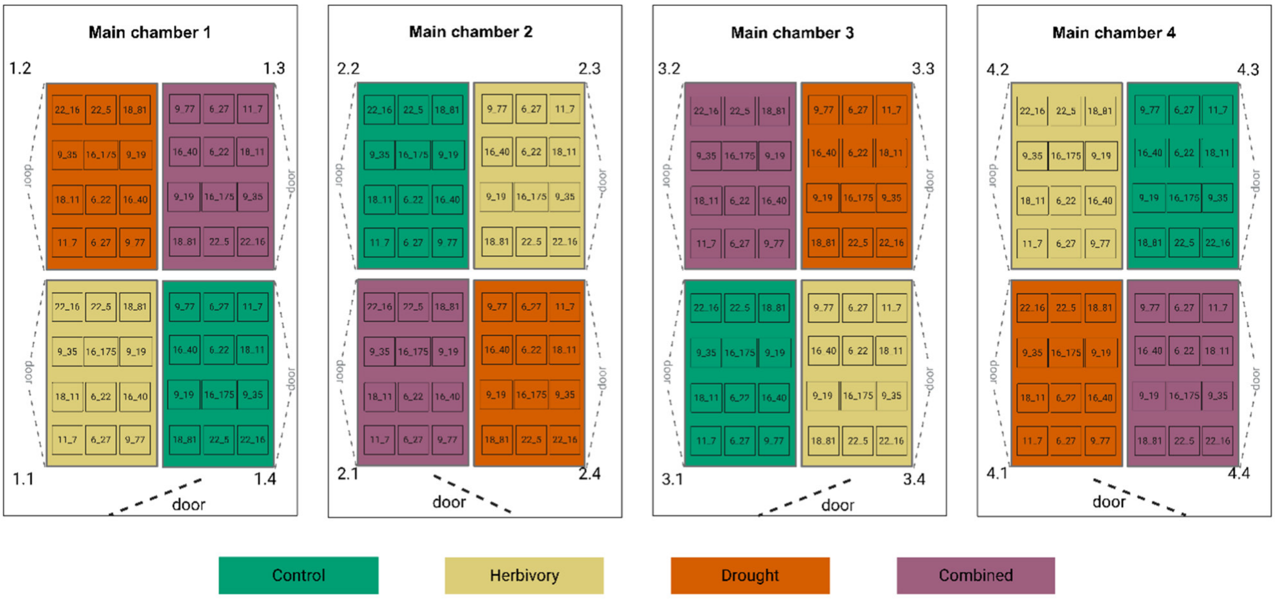

Figure S1. Set-up of *Tanacetum vulgare* clones in experimental chambers. The experimental set-up consisted of four main chambers, with four sub-chambers in each main chamber. Clonal plants experienced one of the four treatments control, drought, insect herbivory and combined challenges (drought and herbivory) in one of four sub-chambers within the main chambers. Clones of one plant individual are indicated by an ID consisting of the maternal origin and the offspring number (for codes see Table S2).

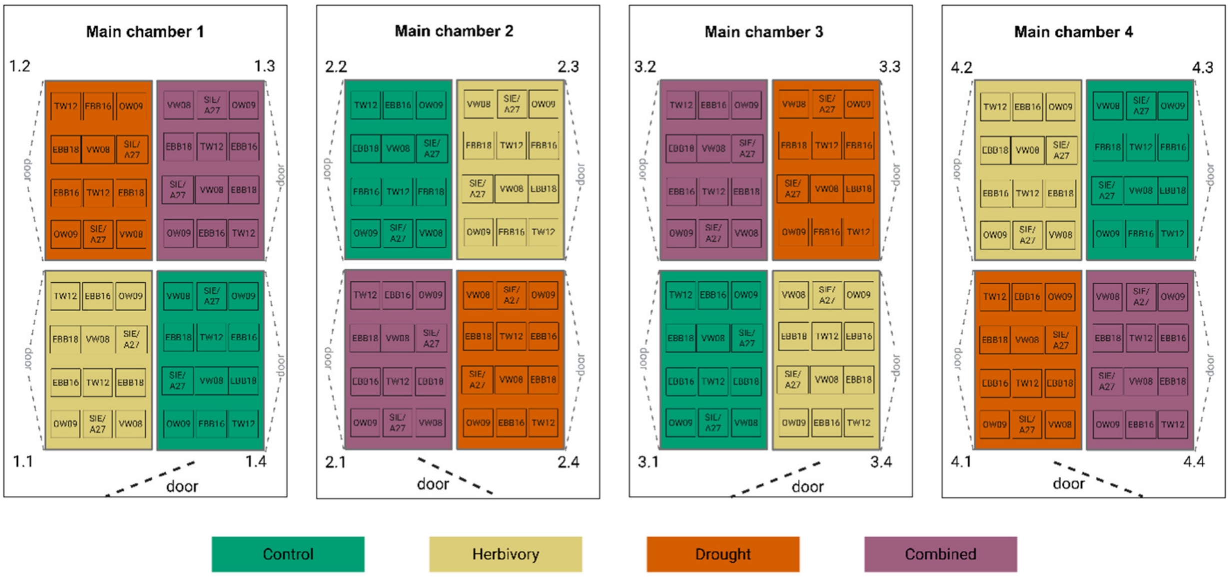

Figure S2. Set-up of *Solanum dulcamara* plants in experimental chambers. The experimental set-up consisted of four main chambers, with four sub-chambers in each main chamber. Clonal plants experienced one of the four treatments control, drought, insect herbivory and combined challenges (drought and herbivory) in one of four sub-chambers within the main chambers. Clones of one plant individual are indicated by an ID (for codes see Table S3).

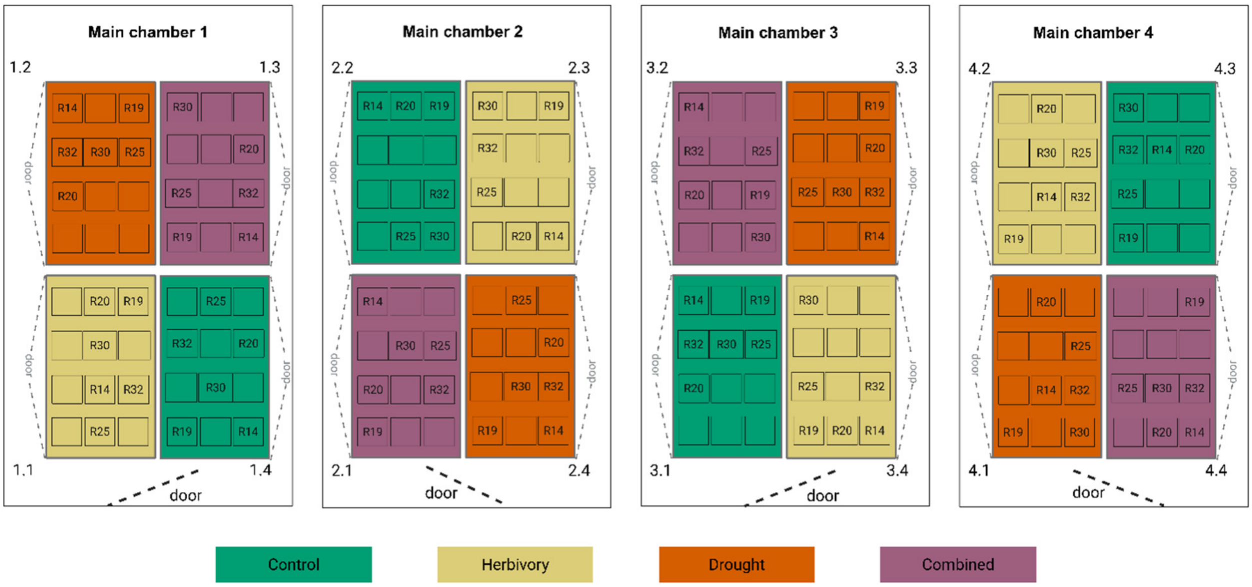

Figure S3. Set-up of *Populus nigra* plants in experimental chambers. The experimental set-up consisted of four main chambers, with four sub-chambers in each main chamber. Clonal plants experienced one of the four treatments control, drought, insect herbivory and combined challenges (drought and herbivory) in one of four sub-chambers within the main chambers. Clones of one plant individual are indicated by an ID (for codes see Table S4).

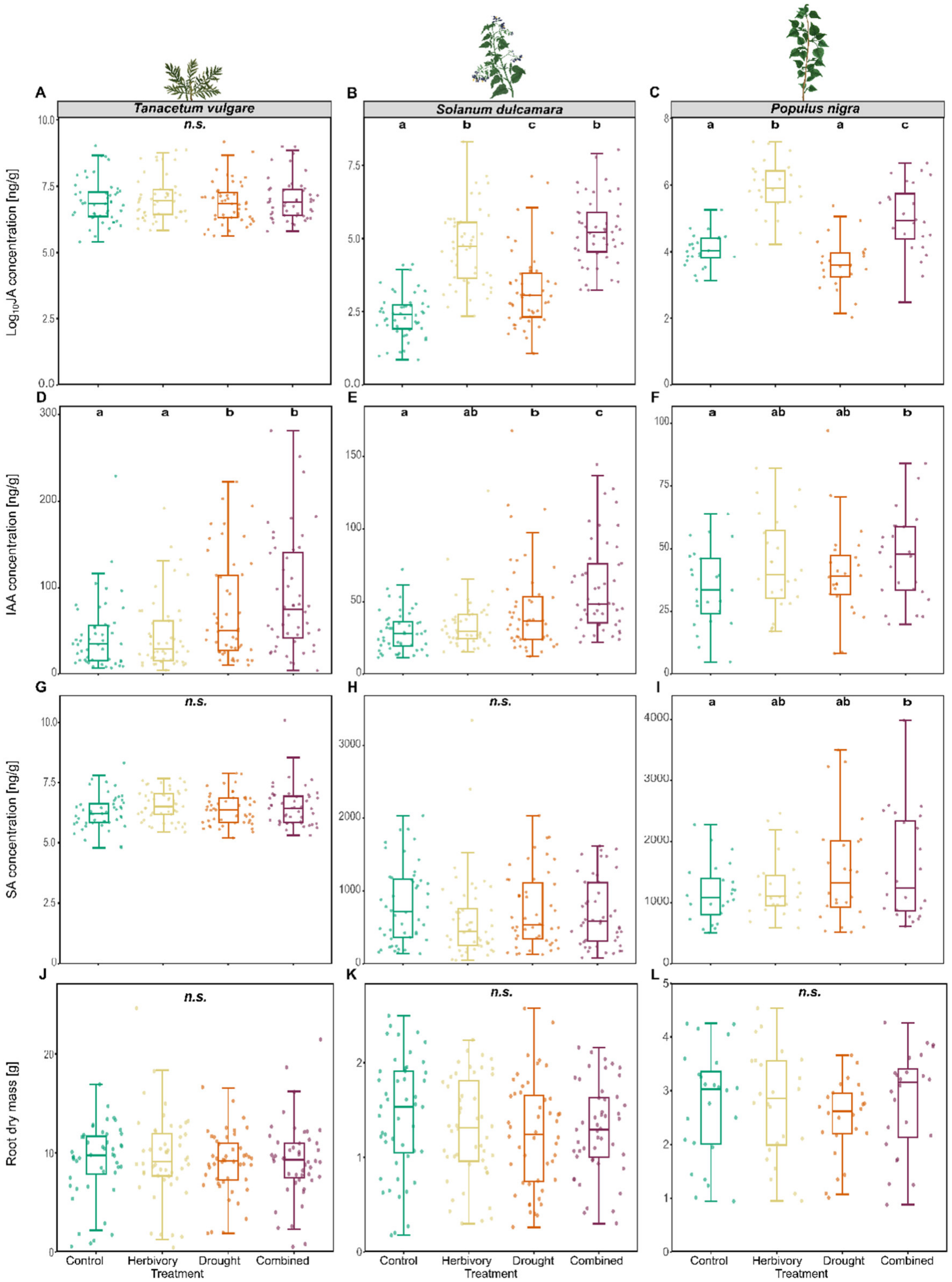

Figure S4. Boxplots of foliar phytohormone concentrations (JA: jasmonic acid; IAA: indole acetate acid; SA: salicylic acid) and root dry mass as morphological trait across treatments (combined: herbivory and drought) of each species, *Tanacetum vulgare* (A, D, G, J), *Solanum dulcamara* (B, E, H, K), and *Populus nigra* (C, F, I, L). Data are presented as boxplots, with medians, interquartile ranges (IQR, boxes), and whiskers extending to the most extreme values with max. 1.5 times the IQR. Individual values are plotted as dots; *n* = 48 per treatment for *T. vulgare* and *S. dulcamara*, n = 24 per treatment for *P. nigra*. Different letters indicate statistically significant differences (F-test, $p < 0.05$); *n.s.*: not significant. Please note the different scaling on the y-axes.

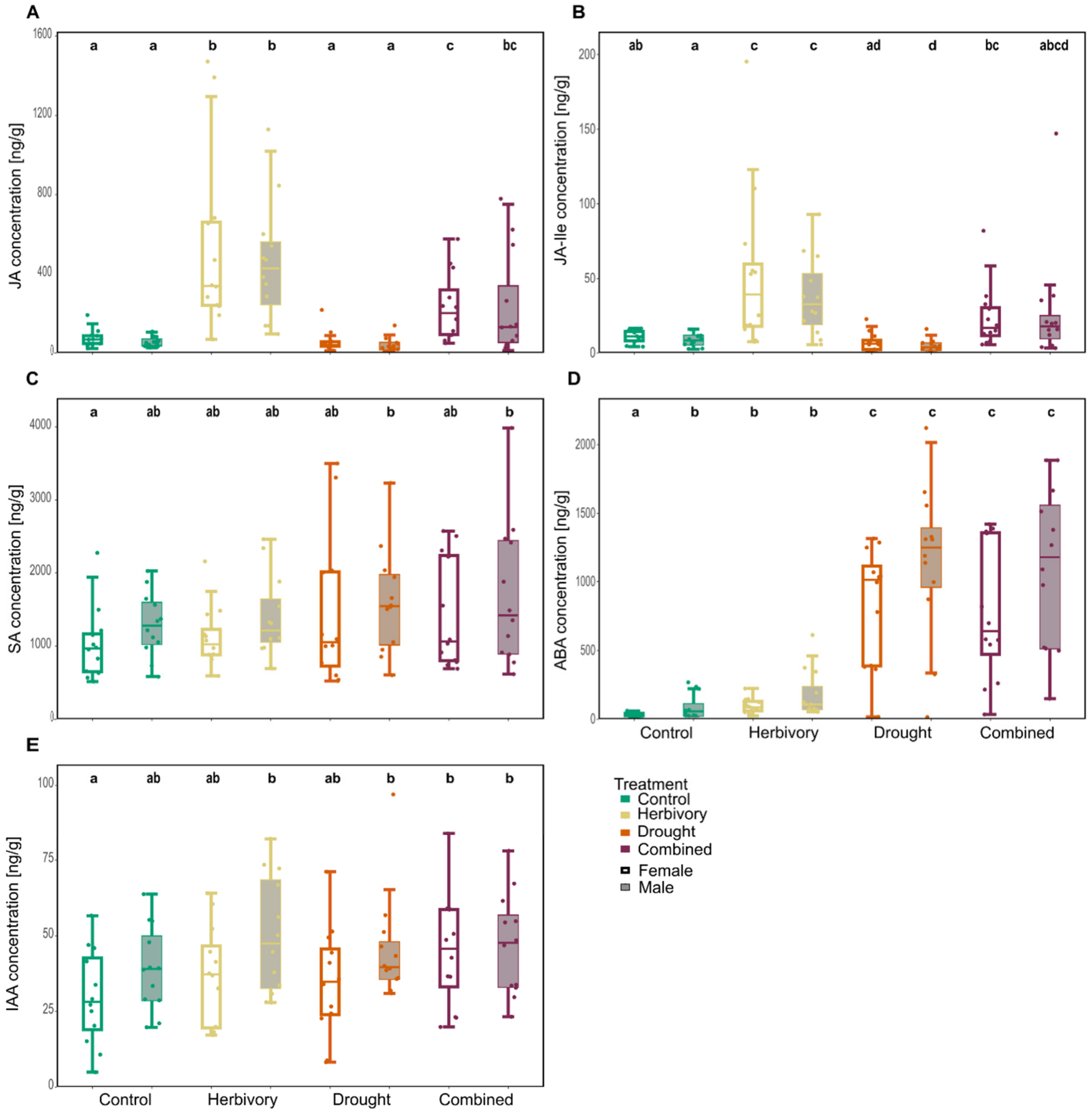

Figure S5. Boxplots of foliar phytohormone concentrations [JA: jasmonic acid (A); JA-Ile: jasmononyl isoleucine (B); SA: salicylic acid (C); ABA: abscisic acid (D); IAA: indole acetate acid (E)) across treatments (combined: herbivory and drought) and sex of *Populus nigra*. Data are presented as boxplots, with medians, interquartile ranges (IQR, boxes), and whiskers extending to the most extreme values with max. 1.5 times the IQR. Individual values are plotted as dots; n = 24 per treatment for *P. nigra*. Different letters indicate statistically significant differences (F-test, $p < 0.05$).

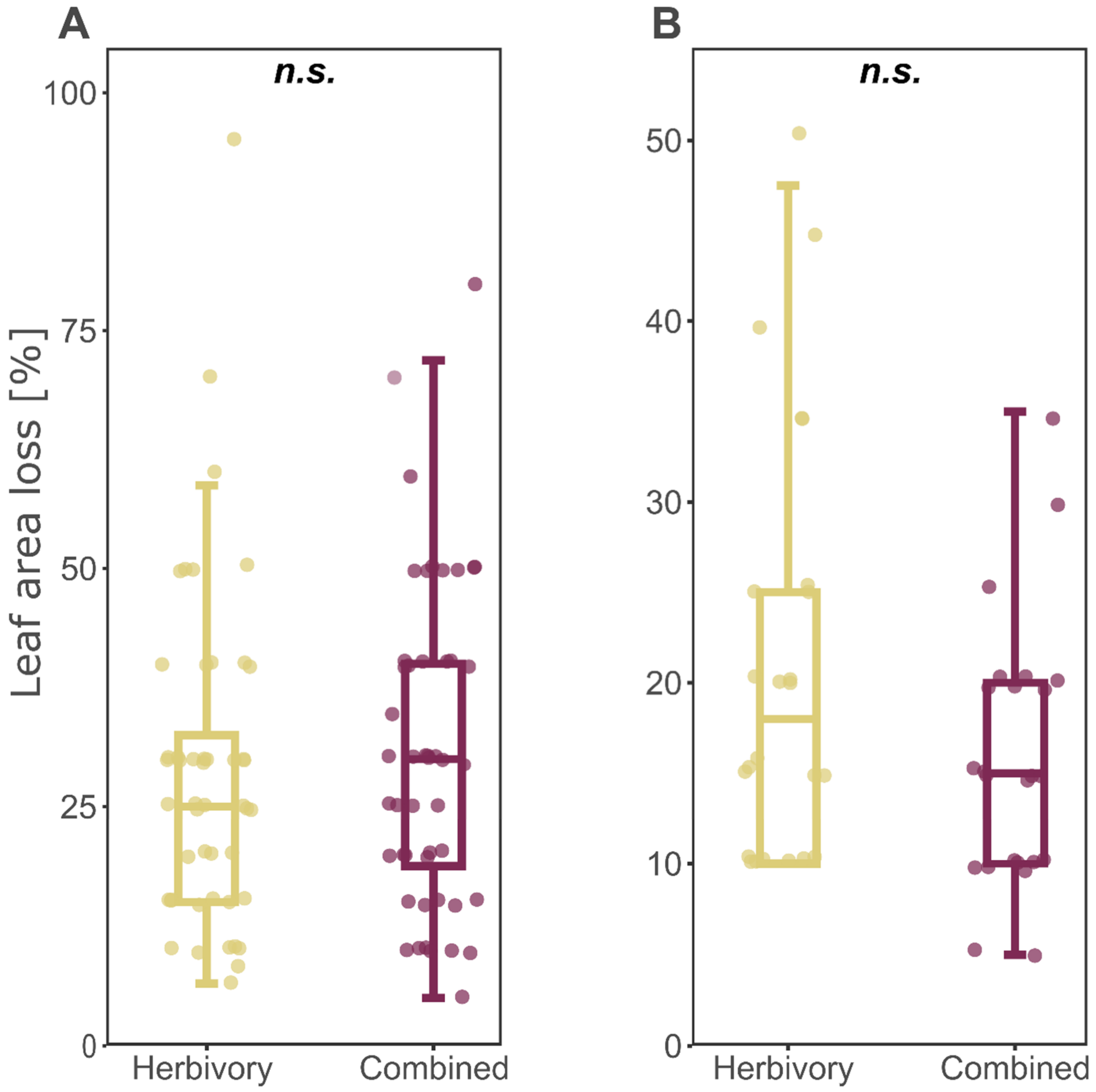

Figure S6. Boxplots of estimated leaf area loss in *Solanum dulcamara* (A), and *Populus nigra* (B) under herbivory or a combined (herbivory and drought) treatment. Observers were trained using ZAX herbivory images until they reached at least 10% estimation accuracy prior to the experiment. Leaf area loss was not assessed for *Tanacetum vulgare* because larvae of *Spodoptera exigua* primarily fed on the petiole rather than the leaf blade. Data are presented as boxplots, with medians, interquartile ranges (IQR, boxes), and whiskers extending to the most extreme values with max. 1.5 times the IQR. Individual values are plotted as dots; $n = 48$ per treatment for *S. dulcamara*, n = 24 per treatment for *P. nigra*. No significant differences were found (*n.s.*, Mann-Whitney U test test, $p > 0.05$).

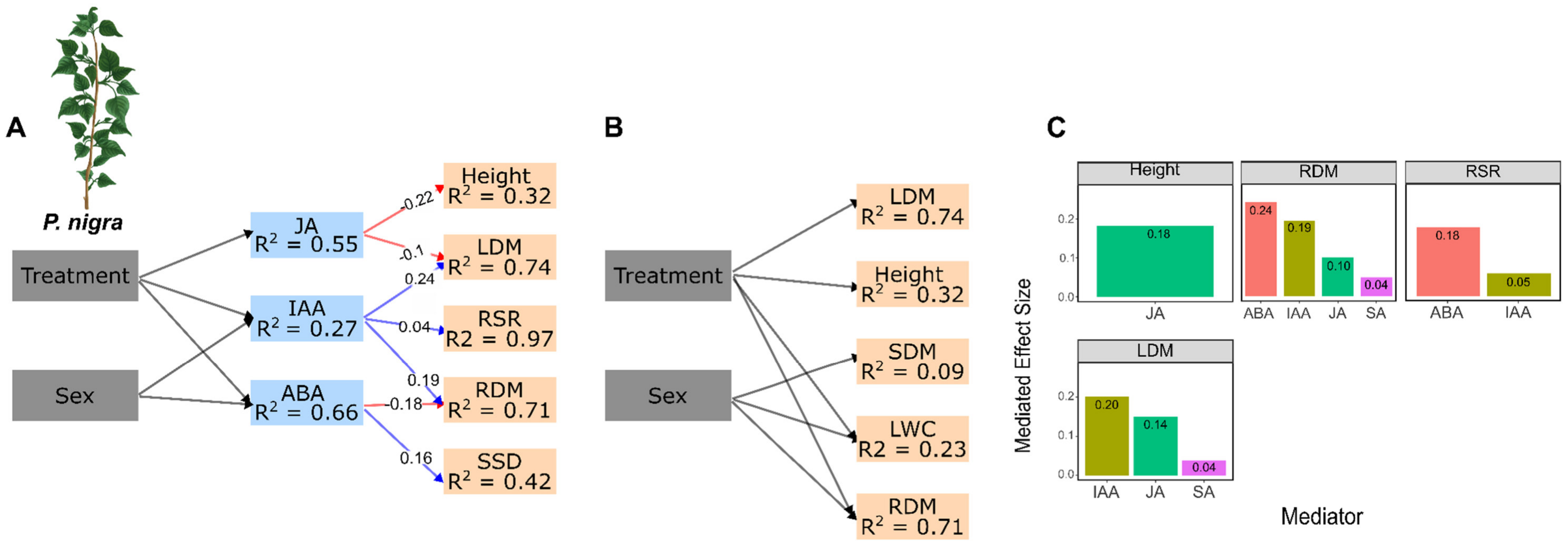

Figure S7. Structural equation models (SEMs) showing how foliar phytohormones regulated morphological responses to treatments in *Populus nigra*. Direct relationships between treatment and sex on foliar phytohormone concentrations, and between phytohormones and morphological traits in *P. nigra* (A). Numbers on each arrow represent standardized effect sizes. Blue arrows indicate positive relationships; red arrows indicate negative ones. Direct effects of treatment and sex on morphological traits in *Populus nigra* (B). The SEM showed imperfect fits, because some paths without biological causes were intentionally excluded (*Populus nigra*: Fisher's C = 94.14, *df* = 66, *p* = 0.01). Coefficients of determination (R^2) for each endogenous variable are shown in the corresponding boxes. For each SEM, the magnitudes of phytohormone mediator effects (i.e., all indirect pathways linking treatment and sex to morphological traits) are shown as bar plots (C: *Populus nigra*). Bootstrapped mediator effects are presented as absolute values to allow comparison of effect strength regardless of direction. The morphological traits are: LN: leaf number; LDM: leaf dry mass; SDM: stem dry mass; RDM: root dry mass; RSR: root-shoot ratio; LWC: leaf water content; SSD: specific stem density.
